# Immunogenicity and safety of intradermal influenza vaccine in immunocompromized patients: a meta-analysis of randomized controlled trials

**DOI:** 10.1186/s12879-015-1161-z

**Published:** 2015-10-14

**Authors:** Claudia Pileggi, Francesca Lotito, Aida Bianco, Carmelo G. A. Nobile, Maria Pavia

**Affiliations:** Department of Health Sciences, University of Catanzaro “Magna Græcia”, Via Tommaso Campanella, 88100 Catanzaro, Italy

**Keywords:** Influenza vaccine, Intradermal, Intramuscular, Immunocompromized patients

## Abstract

**Background:**

The primary influenza prevention strategy is focused on annual vaccination according to the categories identified in the various countries as being at greatest risk of complications. Many studies were conducted in order to demonstrate that intradermal (ID) vaccine formulation represents a promising alternative to conventional intramuscular (IM) formulation, especially in subjects with an impaired immune system. However, there is no consensus whether the efficacy and safety of ID is equivalent to IM in these subjects. Therefore, we performed a meta-analysis of Randomized Controlled Trials (RCT) to compare the immunogenicity and safety of ID and IM influenza vaccines in subjects with a depleted immune system.

**Methods:**

We conducted a search strategy of medical literature published until November 2014 in order to identify RCTs that evaluated the immunogenicity and safety of ID compared with IM influenza vaccines in immunocompromized patients.

**Results:**

We identified a total of 269 citations through research in electronic databases and scanning reference lists. Of these, 6 articles were included in the meta-analysis, for a total of 673 subjects. The seroprotection rate induced by the ID vaccine is comparable to that elicited by the IM vaccine. The overall RR was 1.00 (95 % CI = 0.91 -1.10) for A/H1N1 strain, 1.00 (95 % CI = 0.90-1.12) for A/H3N2 and 0.99 (95 % CI = 0.84 -1.16) for B strain. No significant differences in the occurrence of systemic reactions were detected (17.7 % in the ID group vs 18.2 % in the IM group) with a pooled RR = 1.00 (95 % CI = 0.67 -1.51), whereas ID administration caused significantly more injection site reactions with a mean frequency of 46 % in the ID group compared to 22 % in the IM group, with a pooled RR = 1.89 (95 % CI = 1.40 -2.57).

**Conclusions:**

The ID influenza vaccine has shown a similar immunogenicity and safety to the IM influenza vaccine in immunocompromized patients, and it may be a valid option to increase compliance to influenza vaccination in these populations.

**Electronic supplementary material:**

The online version of this article (doi:10.1186/s12879-015-1161-z) contains supplementary material, which is available to authorized users.

## Background

The primary influenza prevention strategy is focused on annual vaccination according to the categories identified as being at greatest risk of complications. Recently, in the United States, the recommendations for influenza vaccination has been extended to a larger population by the Advisory Committee on Immunization Practices (ACIP) [1, 2], and all subjects aged ≥ 6 months were included.

The common route of influenza vaccine administration is intramuscular (IM) but, more recently, an intradermal (ID) vaccine formulation, licensed in the European Union in February 2009 and by the Food and Drug Administration (FDA) in May 2011, has become available.

Among the several strategies investigated to increase the immunogenicity of influenza vaccine, researchers have focused attention on ID formulation that appears a valid alternative to IM route because this mode of delivery may be advantageous in terms of immunogenicity, dose sparing, greater acceptability among the patients and lower risk of accidental puncture for healthcare workers, because needle-free injection devices can evoke less pain and stress in patients and avoid unsafe injection practices, in line with the World Health Organization (WHO) objectives [3, 4].

Many studies have been conducted in order to demonstrate that the ID influenza vaccine represents a promising alternative to the IM formulation, especially in subjects with a limited vaccination response caused by an impairment of the immune defenses as a result of many different mechanisms, such as treatment with immunosuppressive drugs or HIV infection [5–7]. However, there is no consensus whether the immunogenicity and safety of the ID vaccine is equivalent to the IM formulation in these subjects. Therefore, we performed a meta-analysis of Randomized Controlled Trials (RCT) to compare the immunogenicity and safety of ID and IM influenza vaccine in immunocompromized subjects.

## Methods

### Search strategy for identification of studies

We conducted a search strategy of medical literature published until November 2014 in order to identify RCTs that evaluated the immunogenicity and safety of ID influenza vaccines compared with IM influenza vaccines in immunocompromized patients. The U.S. National Library of Medicine (MEDLINE), Embase, Cochrane Central Register of Controlled Trials, Scopus and Monthly Influenza Bibliography of the National Institute for Medical Research electronic database were searched. Also, we reviewed the reference lists from all retrieved publications and the most recent review articles, in order to identify additional undetected published studies.

The following Medical Subject Heading (MeSH) terms were used individually and in combination in the search: “autoimmune disease”, “cancer”, “comparison”, “HIV infection”, “immunodeficiency disorder”, “immunosuppressive therapy”, “influenza vaccine”, “intradermal administration”, “meta-analysis”, “randomized controlled trials”, “transplant recipients”.

### Inclusion criteria

Articles that met the following criteria were included: (a) RCTs; (b) primary studies; (c) enrollment of all kinds of immunocompromized patients; (d) comparing the immunogenicity and/or safety of the ID vaccine with the standard IM vaccine measuring one or more of the following outcomes: seroprotection and/or seroconversion rate to assess immunogenicity, local reactions and/or general symptoms as safety indicators, according to European Medicines Agency (EMA) criteria; (e) published through November 2014. Trials that compared ID influenza vaccine with IM administration in healthy population, studies that used pandemic vaccine, re-analyses, reviews, letters and abstracts were excluded.

### Assessment of study quality

Two of the authors independently reviewed the studies included in the meta-analysis to appraise the quality of the individual trials using criteria developed by Chalmers et al. [8] and the method of Jadad et al. [9]. Studies were classified as high quality if their score was higher than the median calculated for each quality scale.

### Data extraction

The following data were collected from each study: (a) name of first author, year of publication, and geographic setting; (b) study design; (c) description of intervention in the ID and IM group; (e) number of subjects in each group; f) patient characteristics (age, gender and cause of impairment of the immune system); g) outcome data: 1) percentage of subjects with a post-vaccination titer ≥40 for each strain, referred as the seroprotection rate; 2) percentage of subjects with either a pre-vaccination titer <10 and a post-vaccination titer ≥40, or a fourfold rise in titer from a pre-vaccination titer ≥10, defined as the seroconversion rate; 3) percentage of subjects with at least one injection site reaction (pain, erythema, swelling, pruritus, induration and ecchymosis); 4) percentage of subjects with at least one systemic sign or symptom (fever, myalgia, headache, malaise and shivering).

### Statistical analysis

Risk Ratio (RR) of seroprotection was calculated as the ratio of the percentage of subjects in which seroprotection occurred in those who received ID formulation compared with those who received traditional IM formulation. Similarly, the RR of seroconversion was calculated as the ratio of the percentage of subjects in which seroconversion occurred in ID and IM group. Safety was assessed as the ratio of the percentage of participants that had at least one local and/or general adverse event associated with the ID vaccine compared with those who received the IM vaccine. All meta-analyses were carried out using the DerSimonian and Laird random-effect model [10]. The Mantel-Haenszel method (fixed effects model) [11] was also used. Statistical heterogeneity was assessed using Cochran Q and I^2^ measure; an I^2^ value above 25 % may be considered low heterogeneity, a value above 50 % and 75 % were predefined as moderate and high heterogeneity [12].

### Sensitivity analyses

To explore the reasons for heterogeneity, we performed separate sensitivity analyses by pooling studies that involved subjects with similar characteristics (i.e. having a similar disease that causes immunodeficiency), details of intervention as antigen content, type of ID devices used, and number of injection sites. Also, we performed a meta-analysis to assess the potential effect of the studies’ quality on the results, by combining only studies with Jadad scores greater than or equal to the median (high quality).

Finally, publication bias was explored by searching eventual unpublished RCTs in two clinical trial registries: ClinicalTrials.gov and EudraCT.

All statistical analyses were performed using Stata software program, version 11 (Stata Corporation. College Station, TX).

The reporting of study’s findings was in accordance with the PRISMA statement [13]. PRISMA checklist was used to ensure inclusion of relevant information (See Additional file 1).

## Results

### Study characteristics

**Fig. 1:**
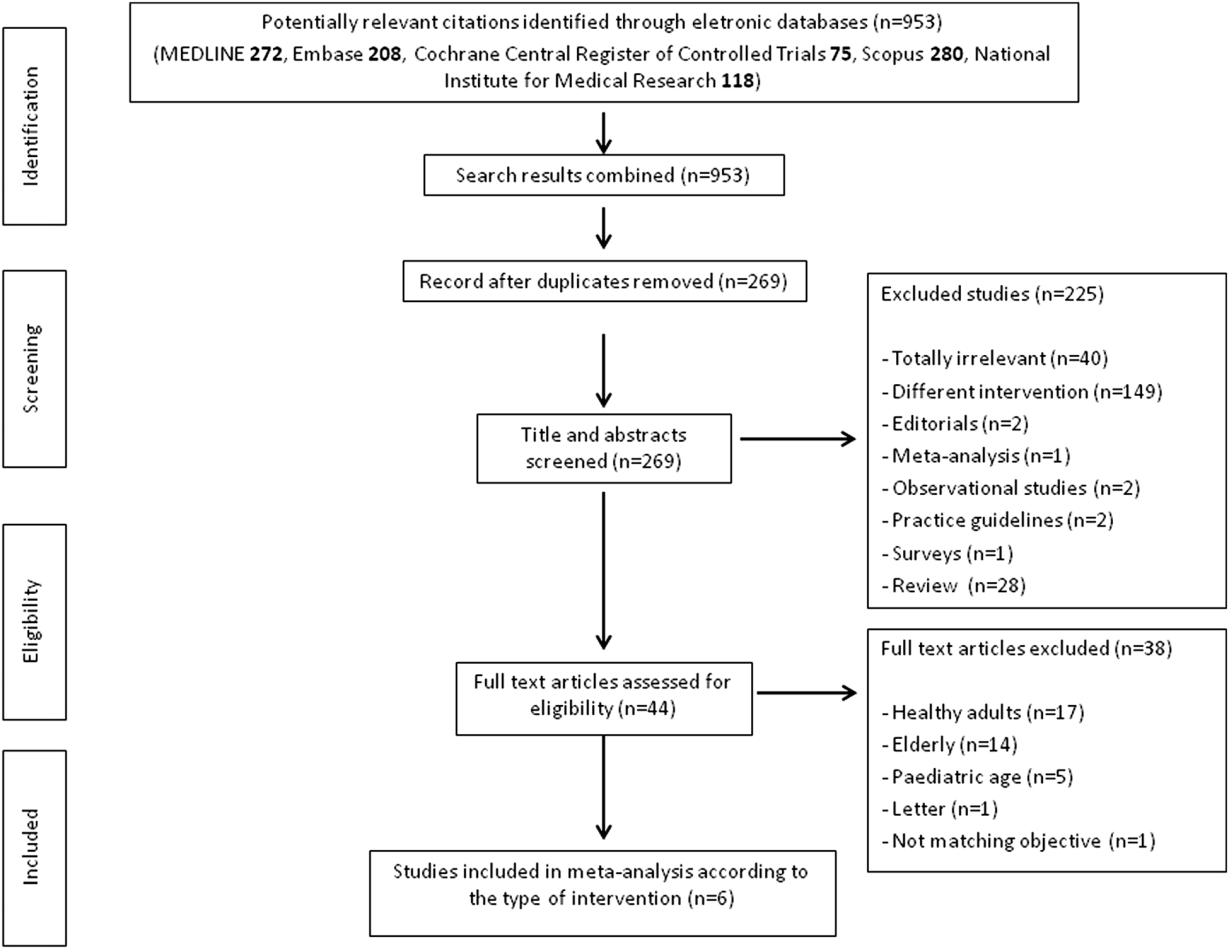
Flow chart of the published trials evaluated for inclusion in the meta-analysis

We identified a total of 269 citations through research in electronic database and scanning references lists. Of these, 6 articles [14–19] met all inclusion criteria and were available for the meta-analysis. A flow diagram describes the reasons for excluding the studies from the meta-analysis (Fig. 1). In particular, only two studies performed in immunocompromized patients were excluded: the first because it was a letter [20] and the second because it verified the role of a booster dose of ID injection in patients vaccinated with a standard dose of IM injection [21].

The main characteristics and extracted data of the included RCTs are summarized in Table 1. The studies were carried out from 2009 until 2013. Patients were 18-77 years old, with a mean age of 48 to 54 in the ID group and 48 to 55 years in the IM group. Males accounted for 50 % to 73 % of participants in the ID group and 47 % to 68 % in the IM group. In 3 studies participants were transplant recipients [16, 17, 19], whilst one other involved patients with solid tumors [15], and another comprised of HIV-infected patients [18] and the remaining study was conducted in patients with different diseases causing immunosuppression [14]. ID trivalent inactivated split-virion vaccine was used in 5 studies [15–19] and in the remaining one a subunit vaccine was used [14]; the antigen content ranged from 3 to 15 μg hemagglutinin (HA)/strain. In the IM group, the traditional vaccination with the same amount of antigen (15 μg HA) of the trivalent inactivated split-virion vaccine was used in all trials. In 2 studies, ID vaccine was administered at two separate injection sites [17, 19]. Two studies used Mantoux technique [14, 15], while in the others the devices employed were microneedles [18] and microinjection systems [16, 17, 19].

**Table 1:** Characteristics of included RCTs on intradermal versus intramuscular administration of influenza vaccine

Authors	Country	Interventions		Units of treatment ID/IM	Immunogenicity						Reported reactions		Population	Quality score	
					Seroprotection			Seroconversion			Injection site^a^	Systemic^b^		Jadad scale	Chalmers scale
					A/H1N1	A/H3N2	B	A/H1N1	A/H3N2	B					
		ID	IM		N (%)	N (%)	N (%)	N (%)	N (%)	N (%)	N (%)	N (%)			
					ID/IM	ID/IM	ID/IM	ID/IM	ID/IM	ID/IM	ID/IM	ID/IM			
L Gelinck et al. 2009 [14]	Netherlands	TIV SU	TIV SU	77/79	49(63.6)/60(75.9)	60(77.9)/58(73.4)	50(64.9)/55(69.6)	NA	NA	NA	Total reactions^c^		Immuno-compromized patients	3/5	0.54
		3 μg HA/strain	15 μg HA/strain								52 % ID group/30 % IM group				
Y Jo et al. 2009 [15]	Korea	TIV SPL	TIV SPL	52/55	50(96.1)/52(94.5)	50(90.1)/54(98.1)	41(78.8)/ 45(81.8)	38(73)/41(74.5)	28(53.8)/24(43.6)	28(53.8)/37(67.3)	10 (19)/3(5.5)^d^	1 (1.9)/2 (3.6)^e^	Patients with solid cancer	2/5	0.32
		7.5 μg HA/strain	15 μg HA/strain												
E Morelon et al. 2010 [16]	France	TIV SPL	TIV SPL	31/31	22(71)/16(52)	16(52)/11(36)	22(71)/19(61)	11(35)/6(19)	11(35)/6(19)	6(19)/6(19)	25 (80.6)/ 15 (48.4)	17 (54.8)/16 (51.6)	Renal transplant patients	3/5	0.45
		15 μg HA/strain	15 μg HA/strain												
O Manuel et al. 2011 [17]	Multicentric^f^	TIV SPL	TIV SPL	41/43	16(39)/ 12(28)	34(83)/ 42(98)	12(29)/ 25(58)	3(7.3)/ 3(7)	2(4.9)/3(7)	3(7.3)/5(11.6)	17 (41.5)/11 (25)	3 (7.3)/7 (15.9)	Lung transplant patients	3/5	0.67
		6^h^ μg HA/strain	15 μg HA/strain												
F Ansaldi et al. 2012 [18]	Italy	TIV SPL	TIV SPL	28/24	22(79)/19(80)	23(82)/19(80)	21(75)/18(76)	14(50)/15(64)	15(54)/14(60)	11(36)/ 8(32)	18 (64.3)/5 (20.8)	6 (21.4)/3 (12.5)^g^	HIV-infected patients	3/5	0.71
		9 μg HA/strain	15 μg HA/strain												
A Baluch et al. 2013 [19]	Canada	TIV SPL	TIV SPL	107/105	76(71)/74(70.5)	75(70.1)/67(63.8)	68(63.6)/55(52.4)	40(37.4)/36(34.3)	31(29)/32(30.5)	23(21.5)/18(17.1)	NA		Transplant patients	3/5	0.75
		9^i^ μg HA/strain	15 μg HA/strain												

ID: Intradermal; IM: Intramuscular; TIV: trivalent inactivated vaccine; HA: hemagglutinin; SPL: split vaccine; SU: subunit vaccine

^a^Pain at injection site, erythema, swelling, pruritus, induration and ecchymosis

^b^Fever, myalgia, headache, malaise and shivering

^c^Frequency of local and systemic adverse reactions calculated on 125 participants that recorded whether or not they had suffered adverse reactions

^d^Referred to swelling that was the most frequent symptom suffered both in ID and IM groups

^e^Referred to fever or myalgia

^f^Canada and Switzerland

^g^Referred to shivering that was the most frequent symptom suffered both in ID and IM groups

^h^Two doses of ID vaccine were delivered for a cumulative dose of 12 μg antigen per strain

^i^Two doses of ID vaccine were delivered for a cumulative dose of 18 μg antigen per strain

Only one trial did not report local and systemic reactions [19], in one study they were pooled [14], in the remaining studies each local and systemic adverse reaction was reported for IM and ID group [15–18]. In 3 studies also subjects with at least one local [16–18] or systemic [15–17] reaction were reported and these values were extracted for the meta-analysis, in others the most frequent local [15] or systemic [18] reaction was extracted. Local reactions ranged from 19 % to 80.6 % in the ID group and from 5.5 % to 48.4 % in the IM group, with an overall frequency of 46 % in the ID group and of 22 % in the IM group. In the ID group the most frequent local reactions were erythema and swelling followed by pruritus, whereas pain was reported only in two studies [15, 16] and the frequency was similar in the ID and IM group. Systemic adverse events ranged from 1.9 % to 54.8 % in the ID group and from 3.6 % to 51.6 % in the IM group (overall frequency 17.7 % ID group vs 18.2 % IM group). The most frequent systemic reported symptoms were shivering and headache.

### Data quality

The mean quality scores of the individual studies using the Chalmers et al. [8] scale ranged from 0.32 to 0.75 (mean = 0.57; median = 0.61). With regard to the Jadad et al. [9] criteria, the mean score was 2.8 (median 3), all trials were classified as open-label and reported a description of withdrawals or dropouts after randomization. According to the Jadad score only one study [15] was below the median, whereas according to the Chalmers score three studies were below the median [14–16]. Scores of individual studies are reported in Table 1.

### Meta-analysis

The results of the meta-analyses that compared the immunogenicity of the ID influenza vaccine with the IM vaccine involving 673 patients for the meta-analysis on seroprotection and 517 patients for that on seroconversion are shown in Table 2.

**Table 2:** Overall and sensitivity analysis results of immunogenicity of intradermal versus intramuscular administration of influenza vaccine

	H1N1				H3N2				B			
SEROPROTECTION	No. studies	No. patients	Overall RR (95 % CI)^a^	Heterogeneity test (p; I^2^%)	No. studies	No. patients	Overall RR (95 % CI)^a^	Heterogeneity test (p; I^2^%)	No. studies	No. patients	Overall RR (95 % CI)^a^	Heterogeneity test (p; I^2^%)
All studies	6	673	1.00 (0.91-1.1)	0.272;21.5	6	673	1.00 (0.9-1.12)	0.042;56.6	6	673	0.99 (0.84-1.16)	0.072;50.5
High quality^b^	5	566	1.01 (0.86-1.18)	0.177; 36.7	5	566	1.02 (0.88-1.19)	0.05;57.9	5	566	0.98 (0.79-1.22)	0.041;59.8
Low quality^b^	1	107	1.02 (0.94-1.11)	-	1	107	0.98 (0.92-1.05)	-	1	107	0.94 (0.79-1.16)	-
Antigen content ≥12 μg	3	358	1.13 (0.9-1.43)	0.248;28.4	3	358	1.03 (0.77-1.39)	0.01;78.4	3	358	0.94 (0.61-1.47)	0.01;78.1
Antigen content ≤ 9 μg	3	315	0.95 (0.8-1.14)	0.067;63	3	315	0.99 (0.93-1.05)	0.368;0.1	3	315	0.96 (0.84-1.09)	0.935;0
One injection	4	377	0.99 (0.86-1.15)	0.136;45.9	4	377	1.04 (0.9-1.2)	0.087;54. 3	4	377	0.98 (0.87-1.11)	0.783;0
Two injections	2	296	1.05 (0.84-1.3)	0.291;10.2	2	296	0.96 (0.72-1.29)	0.015;83.1	2	296	0.8 (0.34-1.99)	0.003;88.8
Mantoux technique	2	263	0.93 (0.71-1.24)	0.012;84	2	263	1.00 (0.89-1.13)	0.177;45.2	2	263	0.95 (0.82-1.1)	0.819;0
Micro injection system	4	410	1.06 (0.92-1.22)	0.378;2.9	4	410	1.02 (0.83-1.26)	0.027;67.4	4	410	0.98 (0.73-1.32)	0.027;67.4
Cause of immunosuppression:												
Transplantation	4	344	1.11 (0.86-1.43)	0.228;30.7	4	344	1.04 (0.8-1.35)	0.22;68.9	4	344	0.91 (0.61-1.36)	0.018;70.2
Other diseases^b^	3	488	0.98 (0.87-1.1)	0.216;34.7	3	488	0.99 (0.93-1.05)	0.465;0	4	488	0.97 (0.86-1.1)	0.98;0
SEROCONVERSION												
All studies	5	517	1.00 (0.84-1.19)	0.532;0	5	517	1.08 (0.86-1.36)	0.569;0	5	517	0.92 (0.72-1.17)	0.578;0
High quality^b^	4	410	1.04 (0.79-1.36)	0.399; 0	4	410	1.00 (0.75-1.34)	0.52;0	4	410	1.13 (0.77-1.66)	0.823;0
Low quality^b^	1	107	0.98 (0.78-1.23)	-	1	107	1.23 (0.83-1.83)	-	1	107	0.80 (0.59-1.09)	-
Antigen content ≥12 μg	3	358	1.17 (0.85-1.62)	0.545;0	3	358	1.06 (0.73-1.55)	0.36;2	3	358	1.11 (0.7-1.75)	0.641;0
Antigen content ≤ 9 μg	2	159	0.95 (0.77-1.16)	0.443;0	2	159	1.1 (0.81-1.45)	0.35;0	2	159	0.85 (0.64-1.13)	0.33;0
One injection	3	221	0.99 (0.74-1.34)	0.242;29.6	3	221	1.16 (0.86-1.57)	0.341;7	3	221	0.86 (0.65-1.13)	0.589;0
Two injections	2	296	1.09 (0.77-1.55)	0.961;0	2	296	0.94 (0.63-1.4)	0.735;0	2	296	1.14 (0.68-1.9)	0.359;0
Mantoux technique	1	107	0.98 (0.78-1.23)	-	1	107	1.23 (0.83-1.83)	-	1	107	0.80 (0.59-1.09)	-
Micro injection system	4	410	1.04 (0.79-1.36)	0.399; 0	4	410	1.00 (0.75-1.34)	0.52;0	4	410	1.13 (0.77-1.66)	0.823;0
Cause of immunosuppression:												
Transplantation	3	358	1.17 (0.85-1.62)	0.545;0	3	358	1.06 (0.73-1.55)	0.36;2	3	358	1.11 (0.7-1.75)	0.641;0
Other diseases^c^	2	159	0.95 (0.77-1.16)	0.443;0	2	159	1.1 (0.81-1.45)	0.35;0	2	159	0.85 (0.64-1.13)	0.33;0

^a^RRs and 95 % CIs were calculated with the DerSimonian and Laird random effect model

^b^Referred to Jadad scores

^c^Solid cancers, HIV infection, rheumatologic disease treated with anti-tumor necrosis factor

**Fig. 2:**
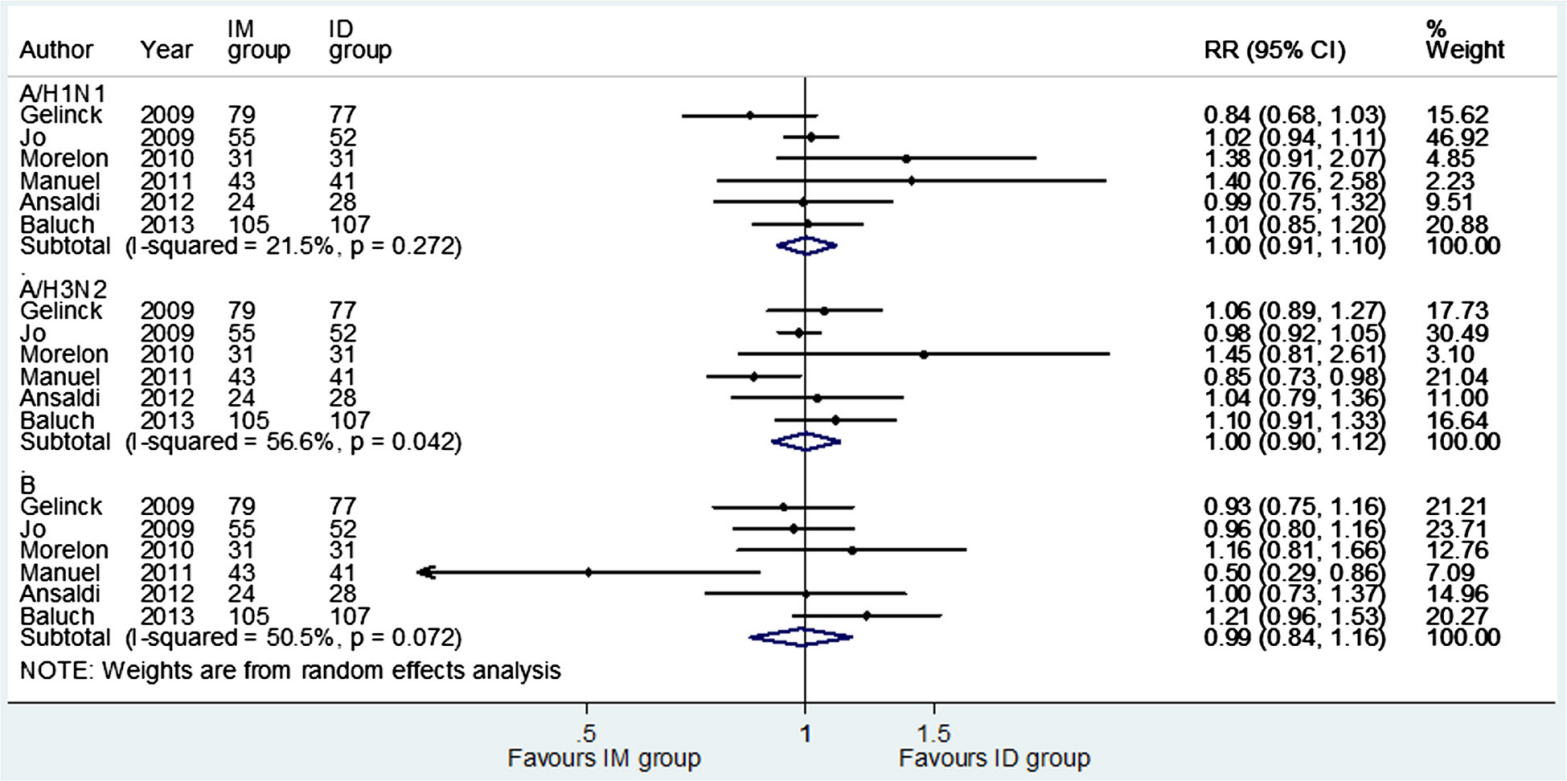
Forest plots of the risk ratio of seroprotection for intradermal compared with intramuscular administration of influenza vaccine according to strains

The seroprotection rate induced by the ID vaccine was comparable to that elicited by the IM vaccine. The overall RR was 1.00 (95 % CI = 0.91-1.10) for A/H1N1 strain, 1.00 (95 % CI = 0.90-1.12) for A/H3N2 and 0.99 (95 % CI = 0.84-1.16) for B strain. The I^2^ statistic test showed a low-moderate heterogeneity (Fig. 2). Similarly, the seroconversion rate achieved with the ID vaccine was found to be equivalent to that of the IM vaccine for each strain (A/H1N1: RR = 1.00, 95 % CI = 0.84-1.19; A/H3N2: RR = 1.08, 95 % CI = 0.86-1.36; B: RR = 0.92, 95 % CI = 0.72-1.17) and no heterogeneity was found.

**Fig. 3:**
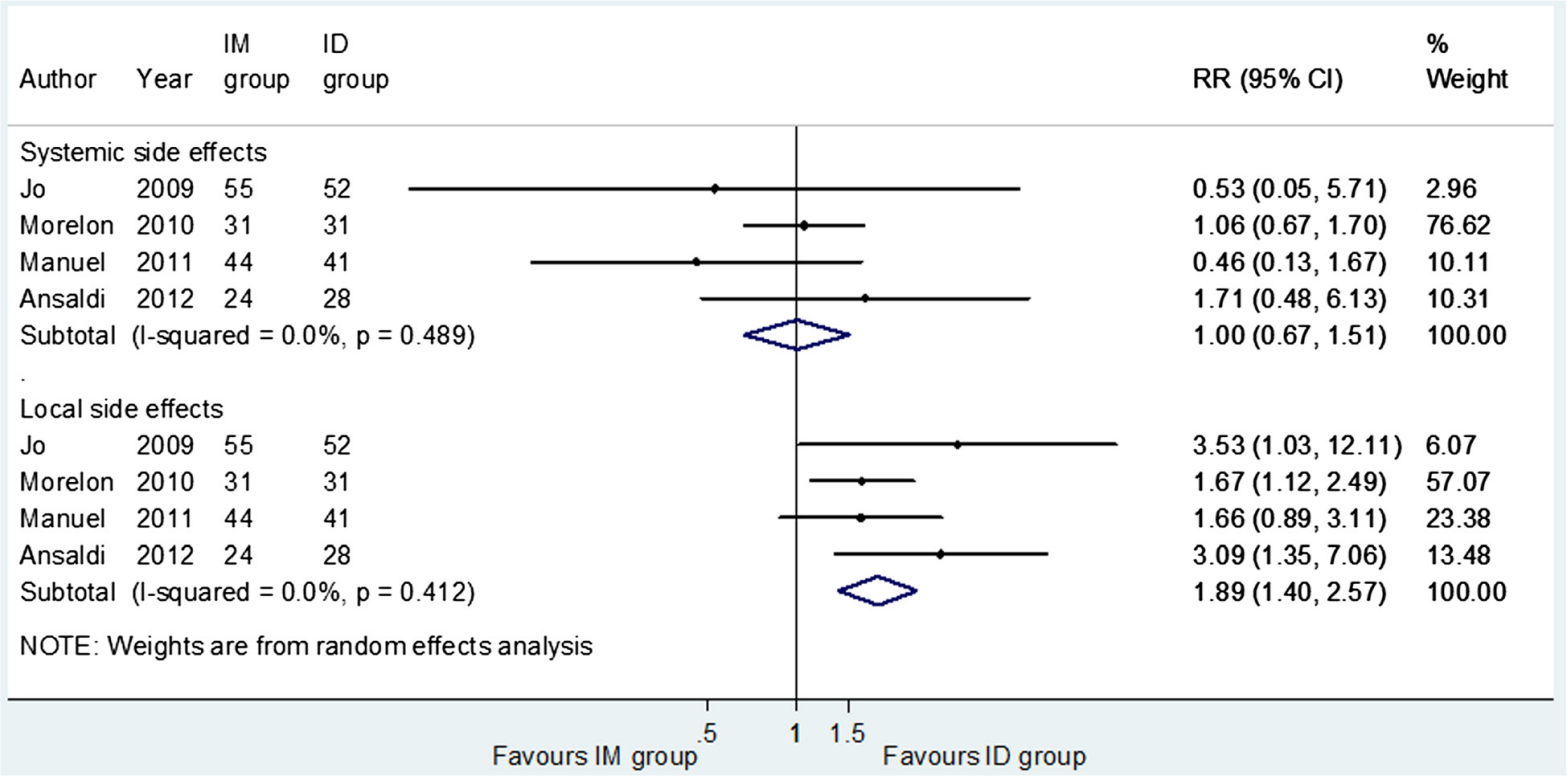
Forest plots of the risk ratio of vaccine safety for intradermal compared with intramuscular administration of influenza vaccine. Systemic side effects: at least one systemic sign or symptom (fever, myalgia, headache, malaise and shivering). Local side effects: at least one injection site reaction (pain, erythema, swelling, pruritus, induration and ecchymosis)

The meta-analyses on vaccine safety (Fig. 3), conducted on 4 trials to estimate injection site and systemic reactions, showed no significant differences in the occurrence of systemic side effects, with a pooled RR = 1.00 (95 % CI = 0.67-1.51). ID administration caused significantly more injection site reactions (RR = 1.89, 95 % CI = 1.40-2.57). The I^2^ statistic test showed no heterogeneity across the trials both for systemic and for local side effects.

### Sensitivity analysis

Pooled analyses after restriction to quality of the studies (high quality, low quality), to antigen content (≤9 μg, ≥12 μg), to type of ID devices used (Mantoux technique, micro injection system), to number of injection sites (one injection, two injections) and to the cause of immunosuppression (transplantation, other diseases) showed that the investigated factors did not substantially influence the findings for all strains (Table 2).

In the sensitivity analyses on seroprotection, heterogeneity disappeared when analyses were stratified by antigen content (≤9 μg) for the A/H3N2 and B strains, by other causes of immunosuppression excluding transplantation for the same strains, and by injection site and by micro injection system for B strain. Results of meta-analysis on seroconversion did not substantially change in all the stratified analyses (Table 2).

All presented data were derived from random effects models; the results of meta-analyses performed using fixed effects models substantially did not change (data not shown).

### Publication bias

To explore publication bias we searched for similar studies registered in ClinicalTrials.gov and EudraCT databases to verify the number and eventually the results of similar unpublished studies. We found all of the studies included in our meta-analysis, whereas three studies on the same topic were not published. Of these, one was not completed (the reasons for which were not specified) and 2 were completed in 2010 and in 2012 but were not published.

## Discussion

The present meta-analysis is the first comparing the immunogenicity and safety of a seasonal ID influenza vaccine with the traditional IM formulation in subjects with an impaired immune system.

The major findings provide support to an equivalence of ID formulation immunogenicity in respect to IM influenza vaccine measured through both seroconversion and seroprotection and consistently demonstrated for the three vaccine strains. The equivalence was reached although antigen content was lower in the ID formulation in most included studies, thus providing advantages in terms of dose sparing.

The findings of this meta-analysis are consistent with those of the meta-analysis by Marra et al. [22] that focused more on the immunogenicity of ID influenza vaccination versus IM formulation in subjects who were ≥18 years of age, but excluded immunocompromized subjects. These Authors, in a meta-analysis that included 13 trials, found no difference in the overall immunogenicity outcomes and, interestingly, they found a significant association between increasing doses of the ID formulation with increasing immunogenicity response, and when the ID antigen content was analogous to that of the IM formulation (15 μg), the ID vaccine appeared to be superior to the IM formulation for all the strains. This is in line with our results demonstrating an equivalence in studies with lower doses of antigen content in the ID formulation.

The efficacy of influenza vaccination has been extensively evaluated in healthy adults and in the elderly, and recently an umbrella meta-analysis has critically reviewed and re-analysed 15 meta-analyses performed in healthy children, in healthy adults, and in the elderly, and those evaluating the pre-pandemic vaccines (H5N1) and the pandemic 2009 (H1N1) vaccines [23]. Although the results are reported in terms of clinical efficacy and therefore not directly comparable to ours, most seasonal influenza vaccines showed both efficacy and effectiveness at an acceptable or high level for laboratory-confirmed cases and of modest magnitude for clinically-confirmed cases.

A number of reviews [24–26], and two meta-analysis [27, 28] have specifically evaluated the role of several vaccinations, including influenza, in immunocompromized patients, since, compared to healthy adults, the immunogenicity of vaccines may be reduced, and the balance between potential benefits and harms of influenza vaccines is hard to establish. In particular, the meta-analyses showed a significant effect in the prevention of influenza-like illness and laboratory confirmed influenza in immunocompromized patients vaccinated with the IM formulation compared to placebo or unvaccinated controls, and no difference in the odds of influenza-like illness compared to vaccinated immunocompetent controls. Less striking results were found for seroconversion and seroprotection rates, and the Authors conclude recommending influenza vaccination in immunocompromized patients. Moreover, in all of these reviews, one of the unresolved issues is the role played by new strategies to improve vaccine response, such as ID administration. Therefore, our results meet the need to clarify the usefulness of ID administration in terms of immunogenicity in patients with weakened immune systems.

Our meta-analysis has highlighted that the ID influenza vaccine in these patients was well tolerated without causing excess harm; indeed, the two modes of administration had an overlapping systemic reactogenity and the ID formulation had a higher amount of local adverse reactions than the IM formulation. However, the higher frequency of injection site reactions in the ID formulation is mostly related to erythema, swelling and pruritus, and these adverse events are not generally a cause of concern for patients [29], while the frequency of pain, the most troublesome symptom, is low and comparable to that caused by IM administration [15, 30]. Thus, based on the results of our meta-analysis, adverse events do not seem to represent a significant safety issue and an obstacle to the acceptability of the ID vaccine. Indeed, previous studies have shown that the acceptability of the ID vaccine is similar [29, 31, 32] or even greater [33] than that of the IM formulation mainly due to the specific injection device. In particular, Foy et al. [33] found that immediately after receiving the ID influenza vaccine, the overall satisfaction rate was 99.6 % versus 88.2 % after the IM vaccine.

Subjects with weakened immune systems are at “high risk” of adverse outcomes as a result of infection with seasonal influenza, as indicated by the WHO [34]. Therefore, any potential initiatives that may enhance the individual willingness to get vaccinated against influenza should be promoted. In this perspective, the advantages provided by the ID influenza vaccine appear useful and successful [32] and support policies oriented to recommendation of influenza vaccination to immunocompromized patients. In this context efforts are strongly needed to improve general practitioners commitment to adherence to vaccination policies, considering their crucial role in the primary health needs of vulnerable patients [35, 36].

### Limitations of the study

The present meta-analysis also has inherent potential limitations. Few studies have examined the immunogenicity of the two influenza vaccines on immunocompromized patients; therefore achieving sufficient statistical power might be difficult, and a cautious approach in the interpretation of results is warranted, especially for stratified analysis, where comparisons were frequently based on three or four trials.

Moreover, the results might have been affected by publication bias, because only published trials have been included and positive studies are more likely than negative ones to be published. Since the low number of studies included in the meta-analysis did not allow any investigation of publication bias through funnel plots or formal tests [37, 38], we tried to explore registries of RCTs to verify the extent of unpublished studies. We retrieved 9 RCTs on the topic of interest and, of these, three were unpublished. Although delayed publication of RCTs on vaccines has been reported [39], the potential for publication bias exists.

Another possible limitation is the heterogeneity among studies, which included subjects with various degrees of immuno-suppression due to different diseases, and to other factors that might have adversely influenced immune responses (eg. age, co-morbidity). Indeed, the heterogeneity was no more significant when analyses were stratified by type of disease. Moreover, studies used different antigen content in the ID vaccine arm, thus reducing the ability of the meta-analysis to identify a recommended dose.

Further research is required to confirm the results of this meta-analysis, to determine the cost-effectiveness of the ID influenza vaccine and to evaluate whether ID administration in the real world, during seasonal vaccination campaign, will be able to increase the adherence to vaccination due to its greater acceptability.

## Conclusions

The ID influenza vaccine has shown a similar immunogenicity and safety to the IM administration in immunocompromized patients, and it may be a valid option to increase compliance to influenza vaccination in these populations.

## Additional file

Additional file 1:
**PRISMA checklist.** (DOC 69 kb)

## Abbreviations

ACIP: Advisory Committee on Immunization Practices
CI: Confidence interval
EMA: European Medicines Agency
FDA: Food and Drug Administration
HA: Hemagglutinin
HIV: Human Immunodeficiency Virus
ID: Intradermal
IM: Intramuscular
RCT: Randomized Controlled Trials
RR: Risk Ratio
WHO: World Health Organization
